# Complement-mediated dialysis reaction during regular hemodialysis treatment: a case report

**DOI:** 10.1186/s13256-024-04365-x

**Published:** 2024-02-08

**Authors:** Ákos Pethő, Karolina Schnabel, László Dézsi

**Affiliations:** 1 Department of Internal Medicine and Oncology, Faculty of Medicine, Semmelweis University, Korányi S. U. 2/a, 1083 Budapest, Hungary; 2 SeroScience Ltd., Budapest, Hungary; 3 Nanomedicine Research and Education Center, Institute of Translational Medicine, Semmelweis University, Budapest, Hungary

**Keywords:** Hemodialysis, Hypersensitivity reactions, Dialysis-related reactions, CARPA, Polysulfone membrane, Pulmonary hypertension

## Abstract

**Background:**

Hemodialysis reactions (HDRs) are similar to complement activation-related pseudo allergy (CARPA), a hypersensitivity reaction that occurs when administering certain (nano)drugs intravenously. The pathomechanism of CARPA was described based on animal experiments. Typical CARPA-like dialysis reactions, which occur at the start of hemodialysis, have been reported using polysulfone dialyzers. However, to our knowledge, this is the first dialysis reaction that occurred towards the end of hemodialysis treatment.

**Case presentation:**

This report describes a 52-year-old Caucasian male patient who had been receiving chronic hemodialysis for 3 years and exhibited a CARPA reaction during his third hour of treatment. Upon activation of the microbubble alarm, the extracorporeal system recirculated for five minutes. Following reconnection, the patient exhibited a drop in systemic blood pressure, chest pain, and dyspnea after five minutes. Symptoms disappeared spontaneously after reducing the speed of the blood pump, placing the patient in a Trendelenburg position, and administering a bolus infusion from the dialysis machine. The remaining dialysis treatment was uneventful.

**Conclusion:**

Numerous case reports about reactions occurring with modern high-efficiency polysulfone dialyzers have been published. However, due to changes in the material structure by the manufacturers, we have not encountered such cases lately. The recently reported increase in thromboxane-B2 and pulmonary arterial pressure and complement activation upon re-infusion of extracorporeal blood following dialysis may explain the reaction observed here.

## Background

Renal replacement therapy, primarily represented by hemodialysis (HD), is a crucial treatment that sustains the lives of an ever-growing population of people worldwide afflicted with stage 5 chronic kidney disease [1]. Despite the progress made in technical advancements and biocompatible materials, HD procedures continue to carry a low risk of acute allergic reactions, also referred to as HD reactions (HDRs), that can occasionally result in life-threatening conditions within minutes of administering the treatment. In recent years, there has been an increasing number of reports documenting hypersensitivity reactions (HSRs) associated with hemodialyzers, which emerged as a new and concerning challenge in the field of nephrology [2–4]. Hemodialysis reaction symptoms (HDRs) are usually characterized by various clinical manifestations such as itching, a burning sensation at the access site, urticaria, flushing, coughing, sneezing, wheezing, abdominal cramps, diarrhea, headache, back and chest pain, nausea, vomiting, fever, and chills. The most common symptoms are typically chest and back pain, dyspnea, nausea, vomiting, and hypotension. These symptoms usually occur within 15–30 min after initiating dialysis. Depending on the severity of the symptoms, discontinuing hemodialysis may or may not be necessary [5]. These reactions and clinical symptoms resemble the experimental findings called Complement Activation-Related Pseudoallergy (CARPA) [6, 7].

## Case presentation

**Fig. 1:**
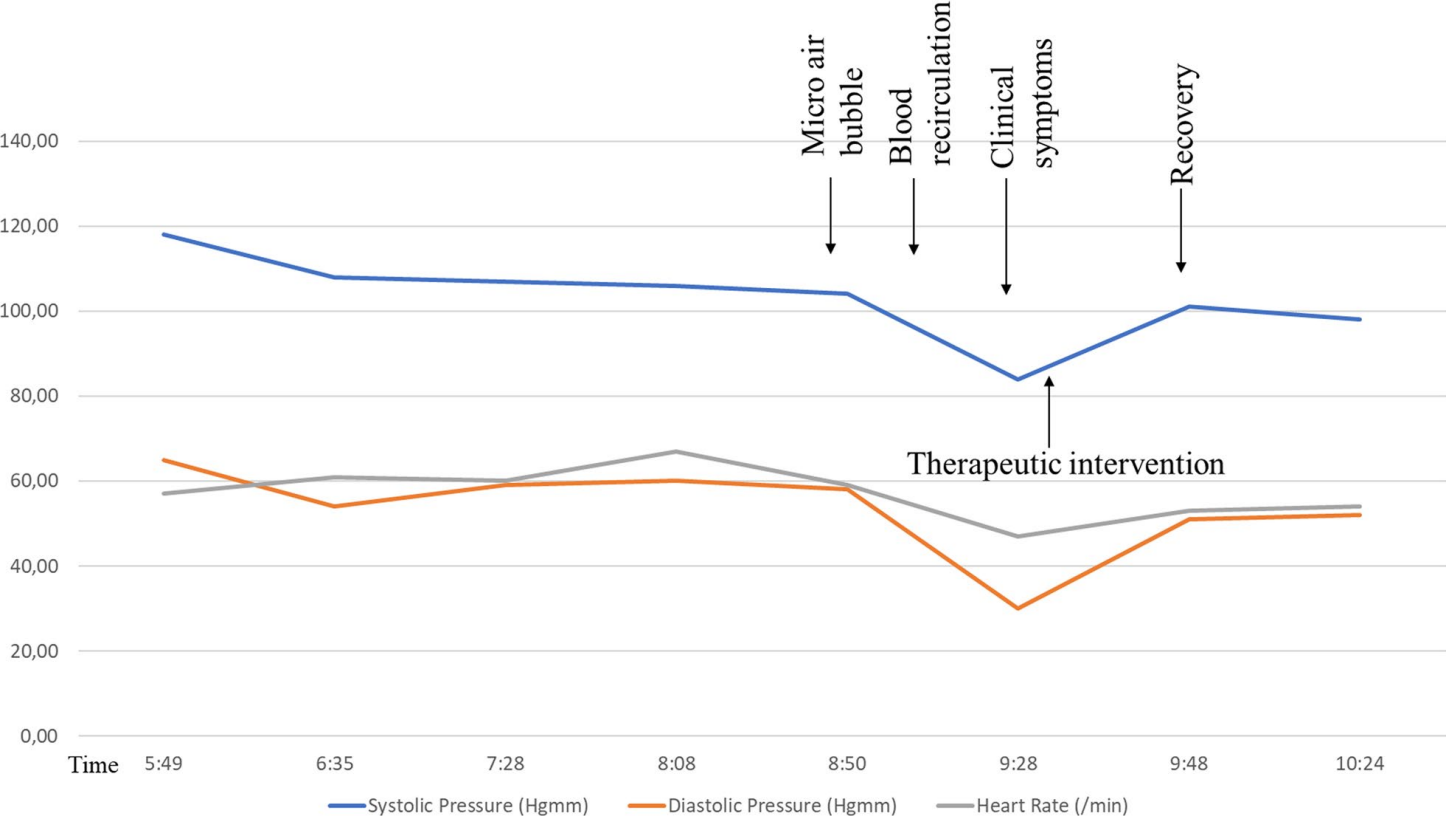
The clinical presentation of the CARPA by our patients. The therapeutic intervention was after the development of clinical symptoms; the ultrafiltration was immediately stopped, the patient was placed in Trendelenburg position, and a bolus infusion of 250 mL from the dialysis machine was given

We present the case of a 52-year-old Caucasian male patient whose kidney failure was caused by ADPKD (Autosomal dominant polycystic kidney disease). Since December 2020, he has received regular hemodialysis (HD) treatment 3 × 4 h weekly. Our patient has no known allergies or allergic and asthmatic diseases. His medication is also the usual, such as tacrolimus, prednisolone, mycophenolate mofetil, cinacalcet, sevelamer bicarbonate, cholecalciferol, allopurinol, pantoprazole, and carvedilol. No reaction or drop in blood pressure occurred during previous HD treatments, which were performed by the 5008 Fresenius Medical Care, Bad Homburg, Germany device. The dialysis treatment prescriptions are standard; the acid concentrates SKF AC-F 313/2 (Potassium^+^ 3 mmol/L, Calcium^2+^ 1.25 mmol/L, Magnesium^2+^: 0.5 mmol/L; glucose: 1 g/L), Natrium^+^ 138 mmol/L, Bicarbonate 29 mmol/L, anticoagulation with Sodium Heparin 3000 IU bolus and 2000 U/h rate. Dialysis treatments were performed through an FX Cordiax 800 filter (Fresenius Medical Care, Bad Homburg, Germany, effective surface 2 m^2^, K_0_A Urea 1365). The vascular access for HD treatment is the left forearm Cimino fistula. The average amount of ultrafiltration is 2000–3000 mL/HD treatment, and the blood pump speed is 450 mL/min. During the third hour of the patient’s 442nd treatment, the dialysis machine detected an air bubble, so the patient’s blood was recirculated following the protocol. The error disappeared in three minutes; the dialysis machine no longer detected the error, so it was possible to reconnect to the patient. Shortly after reconnection and continuation of HD treatment, the patient reported chest pressure and dyspnea. The patient's blood pressure dropped significantly, so the ultrafiltration was immediately stopped, the patient was placed in Trendelenburg position, and a bolus infusion of 250 mL from the dialysis machine was given (Fig. 1). The speed of the blood pump was reduced to 200 mL/min. The patient remained conscious throughout, his condition spontaneously resolved, and 15 min after the start, the initial HD treatment prescription was continued.

## Discussion and conclusion

Hypersensitivity reactions (HSRs) represent a hazard to the clinical implementation of hemodialysis in some patients, yet the mechanism of this phenomenon is poorly understood. A study conducted by Boer et al. observed that various side effects are typically associated with dialysis treatment using PSu/polyethersulfone (PSu/PESu) membranes. These effects include dyspnea, hypotension, hypoxia, bronchospasm, chest pain, pruritus, urticaria, and abdominal symptoms, with a frequency ranging from 22 to 69% of patients within the first 30 min of hemodialysis [8]. Because of these documented HSRs, the surface of the dialyzers was further optimized to eliminate this kind of reaction [9, 10]. Recently, an animal study was published, in which during routine reinfusion (rinsing blood back), a transient increase in pulmonary artery pressure (PAP) and elevation of thromboxane-B2 (TXB2) and complement levels were found. These changes were independent of the type of the dialyzer [11, 12]. Based on the published report, we can conclude that, in our case, a CARPA reaction similar to the HSRs observed during the reinfusion experiments and described with polysulfone dialyzers probably took place. Although we could not confirm the reaction with biochemical markers in our case, the clinical symptoms corresponded to the polysulfone dialyzer reactions described in the literature [8, 13]. In the background of the observed reaction, a mechanism triggered by activated immune cells and mediated by the complement system can be assumed [11, 12]. Based on our hypothesis, the immune cells circulating in the extracorporeal lines and dialyzer were potentially damaged due to hypoxia during the 3-min recirculation. This damage could have resulted in the release of more reactive radicals. After the reconnection, the reactive radicals, activated immune cells, and terminal complement were rinsed back, leading to the clinical symptoms. In all such cases when the patient's blood has to be recirculated due to a technical problem, the patient's vital parameters should be closely monitored after reconnection.

The possibility of additional embolization caused by microbubbles was excluded as the dialysis machine did not produce an alarm message before continuing with the HD treatment. To the best of our knowledge, this is the first such case report presenting HSRs towards the end of HD treatment.

## Data Availability

Data sharing does not apply to this article as no datasets were generated or analyzed during the current study.  Aggregate patient data from the authors upon reasonable request; patients are unidentifiable.
